# Integrity of chromatin and replicating DNA in nuclei released from fission yeast by semi-automated grinding in liquid nitrogen

**DOI:** 10.1186/1756-0500-4-499

**Published:** 2011-11-16

**Authors:** Robert M Givens, Larry D Mesner, Joyce L Hamlin, Michael J Buck, Joel A Huberman

**Affiliations:** 1 Department of Molecular and Cellular Biology, Roswell Park Cancer Institute, Buffalo, NY 14263, USA; 2 Department of Biochemistry and Molecular Genetics, University of Virginia School of Medicine, Charlottesville, VA 22908, USA; 3 Department of Biochemistry and Center of Excellence in Bioinformatics and Life Sciences, SUNY at Buffalo, 701 Ellicott St., Buffalo, NY 14203, USA

## Abstract

**Background:**

Studies of nuclear function in many organisms, especially those with tough cell walls, are limited by lack of availability of simple, economical methods for large-scale preparation of clean, undamaged nuclei.

**Findings:**

Here we present a useful method for nuclear isolation from the important model organism, the fission yeast, *Schizosaccharomyces pombe*. To preserve *in vivo *molecular configurations, we flash-froze the yeast cells in liquid nitrogen. Then we broke their tough cell walls, without damaging their nuclei, by grinding in a precision-controlled motorized mortar-and-pestle apparatus. The cryo-ground cells were resuspended and thawed in a buffer designed to preserve nuclear morphology, and the nuclei were enriched by differential centrifugation. The washed nuclei were free from contaminating nucleases and have proven well-suited as starting material for genome-wide chromatin analysis and for preparation of fragile DNA replication intermediates.

**Conclusions:**

We have developed a simple, reproducible, economical procedure for large-scale preparation of endogenous-nuclease-free, morphologically intact nuclei from fission yeast. With appropriate modifications, this procedure may well prove useful for isolation of nuclei from other organisms with, or without, tough cell walls.

## Background

Studying the proteins, RNA or DNA of the eukaryotic cell nucleus requires physically separating the nucleus from other cellular components. In yeasts and other organisms with tough cell walls, this necessitates breaching the cell wall in such a way that the nucleus remains undamaged [1,2]. A common approach is to use lytic enzymes to digest the cell wall until it fails structurally, as indicated by the cell defaulting to a spherical shape. The plasma membranes of these "spheroplasts" can then easily be permeabilized using detergents. Although spheroplasts remain viable and can recover fully in isotonic buffers, spheroplasting entails prolonged nutrient deprivation and other stresses that could affect nuclear chemistry and structure. The enzymes used in spheroplasting are also relatively expensive, strain- and growth-stage-dependent in efficacy, and have historically been subject to variable quality and availability [3], thus complicating the logistics of large-scale and long-term investigations.

Nuclei from yeast cells broken in aqueous suspension by agitation with glass beads, a common alternative to spheroplasting, are at risk of disruption by mechanical and fluid shear, adventitious biological remodeling and dissociation or degradation of their components. Degradation is of particular concern in cell lysates of the fission yeast, *Schizosaccharomyces pombe*, which often exhibit high levels of endogenous, detergent-stimulated nuclease activity [2]. Furthermore, the results of this method generally vary with both the agitation mechanism used and individual technique.

To protect the integrity of inter-molecular interactions during either procedure, yeast cells are often treated prior to harvest with covalent cross-linking agents such as formaldehyde, thereby fixing many protein-protein and protein-nucleic-acid contacts in place [4-7]. In some cases, however, investigators may wish to avoid the use of chemical fixatives. We therefore wanted to develop large-scale nuclear isolation conditions that would retain as much *in vivo *structure as possible, even in the absence of cross-linking.

We met this challenge by adapting previously-described methods [1,8,9] based on flash-freezing and grinding the yeast cells in -196°C liquid nitrogen ("cryo-grinding"). Here we provide a detailed description of the economical, semi-automated procedure by which we reproducibly obtained large quantities of morphologically intact nuclei from fission yeast cells. In addition, we provide details of our protocols for milligram-scale recovery of high-molecular-weight nuclear DNA bearing intact replication intermediates (RIs), for MNase digestion of chromatin within detergent-permeabilized nuclei, and for gel purification of mononucleosome-sized DNA fragments.

We also present confirmation of the quality of the nuclear preparations resulting from our technique. This includes phase-contrast and fluorescence microscopy, two-dimensional electrophoretic examination of DNA replication intermediate (RI) integrity, and demonstration of accurate nucleosome positioning.

## Methods

### Strains, growth and chemical fixing of fission yeast cells

*Schizosaccharomyces pombe *strain 972 h- was used for DNA RI isolation, and a D18 [10] strain bearing the plasmid pLS-LCS1+2+3 [11] was used for chromatin analysis. The plasmid was transfected into the cells for other purposes and was not relevant to the experiments described here. The cells were grown in 2.5 l or less of minimal medium (EMM [12])/4-l flask at 25°C with 200 rotations per minute (rpm) agitation to either log phase (5-8 × 10^6 ^cells/ml) or stationary phase (1-2 × 10^8 ^cells/ml), as required. Culture growth phase was documented by DAPI-fluorescence, phase-contrast microscopy. Chemically-fixed (cross-linked) samples for chromatin analysis were prepared by adding 37% w/w formaldehyde (Fisher Scientific # F79-500) to a final concentration of 1.5%, then allowing incubation to continue with rotary agitation for 15 min. The formaldehyde reaction was quenched by addition of glycine (SIGMA #G-7126) to a final concentration of 125 mM (accounting for the added volume) with continuing agitation for a minimum of 20 min (min).

Fission yeast cells for DNA replication studies were metabolically fixed by adding sodium azide to log-phase cultures to a final concentration of 0.1%. The flasks were rapidly mixed, then chilled on ice.

### Flash freezing cells

**Figure 1:**
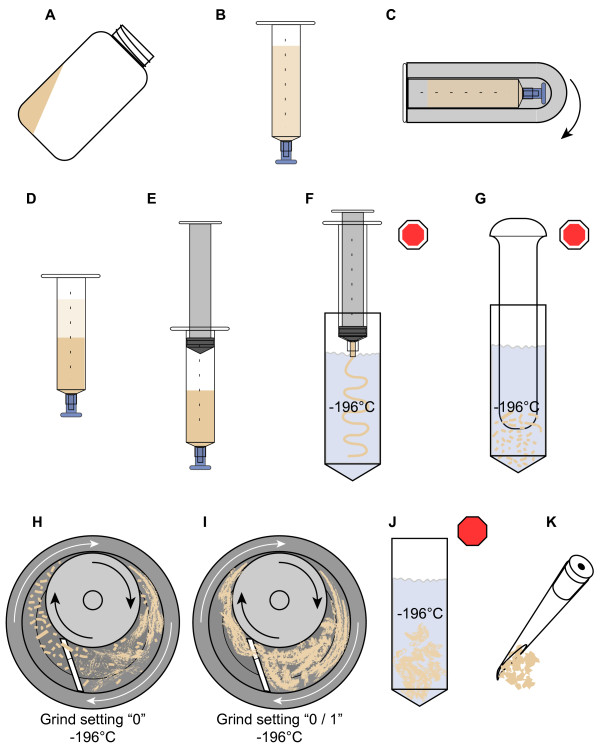
**Flash freezing and semi-automated precision grinding of fission yeast in liquid nitrogen**. **(a) **Pellet of fission yeast cells (beige) in a centrifuge bottle. The cells were harvested by centrifugation, washed and pooled in water, then re-pelleted and drained. **(b) **Pellet resuspended using minimal volume of INCA buffer (see text), then transferred to tip-capped syringe. **(c) **Syringe inserted into tube adapter (gray) and centrifuged in horizontal swinging-bucket rotor. Arrow indicates horizontal rotation. **(d) **After centrifugation, the total (upper boundary of light-beige supernatant) and pellet (upper boundary of dark-beige cell pellet) volumes were recorded by direct reading from the syringe scale. **(e) **Supernatant removed and plunger re-installed. **(f) **Tip cap removed, and packed fission yeast swiftly extruded into liquid nitrogen (blue) as a continuous bead or "noodle". **(g) **Noodle broken into 3- to 7-mm pieces in liquid nitrogen. **(h) **Pieces loaded into pre-cooled (liquid nitrogen) motorized precision mortar grinder and ground in liquid nitrogen at the minimum pressure setting ("0"). Arrows indicate directions of rotation of the mortar and contents (white arrows) and pestle (black arrows). **(i) **When slurry of ground material appears homogenous, pressure setting is raised to pre-determined optimum ("0/1" in our case) until release of intact nuclei, as determined by fluorescence microscopy, is maximized. **(j) **Cryo-ground cells transferred to storage container for evaporation of liquid nitrogen at -80°C. *Octagons *denote steps at which samples may be safely stored at -80°C (initially only loosely capped to allow venting of the evaporating nitrogen). White-bordered octagons: -80°C storage optional; solid red octagon: storage required. **(k) **200-μl pipette tip bevel-cut and bent for collecting samples of mortar contents for microscopy during grinding

Cells (1 × 10^10 ^log phase, 4 × 10^10 ^stationary phase) were harvested by centrifugation in an SLA-3000 rotor (Sorvall) at 6000 rpm, 25°C for 6 min. The centrifugation and wash temperature for azide-treated cells used in RI analysis was 0°C. The pellets were re-suspended and pooled in a total of 100 ml (per 1 × 10^10^-4 × 10^10 ^cells) of sterile distilled water at 25°C (0°C for RI preps). The rinsed cells were re-pelleted as above (see Figure 1a), then suspended in 7-8 ml of water (25°C; 0°C for RI preps), the minimum amount necessary to allow quick, complete loading into the barrels of tip-capped 12-ml polypropylene Luer-lock syringes (Kendall Monoject #1181200777) via serological pipette (Figure 1b). The filled syringe(s) was fitted into a clean adapter sleeve (Corning #8441), then centrifuged horizontally in an HB-6 rotor (Sorvall) at 4,500 rpm, 25°C for 6 min (Figure 1c). The clear supernatant (Figure 1d) was carefully decanted after recording the volume of the firm pellet from the syringe scale. The syringe plunger was then reinserted (Figure 1e), followed by removal of the tip cap and the rapid, but even, extrusion of the paste-like contents as a continuous bead or "noodle" into liquid nitrogen (Figure 1f) held in a tared, screw-top polypropylene container. Care was necessary both to prevent the extruded noodle from piling up on the surface of the liquid nitrogen or freezing prematurely in the syringe tip. The container was then loosely closed and placed in a freezer held at -80° to allow the nitrogen to boil away gradually while leaving the noodles hard-frozen. Once the liquid nitrogen was completely evaporated, the container was sealed and the noodles stored at -80°C for up to 1 year.

### Disruption of flash-frozen cells by cryo-grinding

The walls of the frozen cells were disrupted using an adaptation of previously-described methods [8,9] employing the Retsch RM100 motorized mortar-grinder (http://www.retsch.com/; the RM100 has recently been further improved and upgraded to the RM200 model) with stainless steel grinding set. The tightly-sealed container bearing the cell "noodles" (previous paragraph) was placed in liquid nitrogen in a Dewar Flask for transport from the freezer to the work area. The container and contents were quickly weighed, then returned to the liquid nitrogen, and the mass of the noodles was recorded.

The RM100 pestle-pressure settings described in the following paragraph were determined empirically, in simple preliminary experiments, to provide optimum liberation of intact nuclei from frozen fission yeast cells. The excellent results obtained with these settings proved reproducible over multiple preparations.

Preparation of the RM100 began with setting the timer to "infinity" and the pestle pressure to "0", followed by the proper seating of the stainless-steel mortar bowl onto the drive platform. Although it is sometimes recommended that the mortar bowl be pre-cooled in a freezer [8], we found that this often leads to seating difficulties and build-up of frost, which can both dilute the final product and interfere with grinding. Once the mortar was in place, it was filled with liquid nitrogen. The liquid nitrogen was replenished as it boiled away, keeping the level high to exclude water frost. When the boiling of the liquid nitrogen stabilized at a moderate to low rate, the grinder lid, bearing the matching, previously-aligned pestle and scraper, was quickly closed and locked (see the manufacturer's instructions for details), and the "start" switch was pressed immediately. Note: if there is too long a delay between contact of the pestle with the liquid nitrogen and the start of rotation, the pestle bearings will seize up and overload the motor. The liquid nitrogen level was maintained at the "shoulder" of the pestle through the opened window in the lid using a polypropylene beaker. When the boiling rate in the mortar-grinder again stabilized, indicating full cooling of the grinding set, the sample noodles were prepared for loading. This was done by adding enough liquid nitrogen to the storage container to cover the noodles, which were then quickly broken up into 3- to 7-mm pieces using a liquid-nitrogen-chilled conventional ceramic pestle (Figure 1g). These pieces, in liquid nitrogen, were then poured into the mortar bowl through the grinder's loading window. From this point on, the liquid nitrogen level was only maintained to the top edge of the scraper. Addition of liquid nitrogen was used to wash any adhering sample from the wall of the mortar or pestle surfaces that accumulated above the level of the main pool. The sample was initially ground at pestle-pressure setting "0" (Figure 1h) until it was of a uniform pasty consistency (e.g., 7.5 min for the 2-g Log-phase Unfixed Narrow D18 sample [LUN; Additional File 1C]; 15 min for the 5-g Stationary-phase Unfixed Narrow D18 sample [SUN; Additional File 1C]). The pressure setting was then increased to the "0/1" position (previously determined empirically for the strains used), and grinding was continued (Figure 1i) for another 7.5 min for the 2-g sample and 15 min for the 5-g sample, until adequate disruption of cell walls was attained as confirmed by DAPI-fluorescence microscopy (see next section). For the 10.5-g 972 h- sample, the grinding times used were 20 min at setting "0" and 16 min at "0/1".

Once grinding was judged complete, the "stop" switch was pressed, and the lid was opened, allowing the mortar bowl to be removed and propped securely at a 35-45° angle to encourage the slurry of ground cells and liquid nitrogen to gather in a tight pool for easier transfer to the storage container(s). During transfer, the slurry in the mortar bowl was kept wet with liquid nitrogen. For storage containers, we used tared, screw-top 50-ml polypropylene tubes, each of which was supported upright in a liquid-nitrogen-filled polypropylene beaker and was itself partially filled with liquid nitrogen (Figure 1j). To avoid scratching the precision-ground mortar bowl, we used a plastic teaspoon, pre-chilled in liquid nitrogen, to transfer the slurry. Once recovery of ground cells was complete, the lid was screwed loosely onto the storage tube, which was then transferred quickly, to avoid boiling over of its contents, to a Dewar flask holding just enough liquid nitrogen to immerse the tube to within 1 cm of its top. The Dewar flask was then placed in a -80°C freezer until the liquid nitrogen boiled away completely, after which the storage tube lid was sealed. The ground cells were kept at -80°C until used.

For later samples (log, fixed D18), we used an improvement of the final step, which was designed to facilitate dispersion of the ground cells into the aqueous buffer used in later steps. "In-Nucleo Chromatin Analysis" (INCA) buffer consists of 1.2 M Sorbitol, 100 mM NaCl, 50 mM HEPES-pH 7.4, 5 mM CaCl_2_, 2 mM MgCl_2_, with 1 mM 2-mercaptoethanol and 0.5 mM spermidine (Fluka BioChemika #85558) added immediately prior to use, all at 0°C. INCA buffer was designed to preserve nuclear morphology under aqueous conditions. For the improved final step, 5 ml of INCA buffer per gram (g) ground cells were sprayed through a fine-mist atomizer into 900-1000 ml of rapidly stirred liquid nitrogen in a deep polypropylene beaker, at 3-4 s intervals between additions, so as to produce flash-frozen, presumably compositionally homogeneous, mini-beads of INCA buffer, which were then cryo-ground stepwise in the RM100 at sequential pressure settings 0, 2 and 5 to a uniformly smooth paste. The pressure setting was then reduced to 0/1, and the desired amount of ground cells, suspended in liquid nitrogen (from the preceding paragraph), was added to the rotating mortar bowl and allowed to mix with the buffer cryo-paste until homogeneous. This "cryo-mix" was then collected and stored as described in the preceding paragraph.

Initially it is best to have an assistant to either monitor and maintain the liquid nitrogen levels in the operating grinder or to perform the diagnostic microscopy. Once the grinding protocol has been optimized, the microscopy of time-point aliquots becomes merely confirmatory and can be deferred until the procedure is completed, thus allowing a single investigator to execute the method.

### Assessment of nuclear release and integrity by phase-contrast and DAPI-fluorescence microscopy

For fluorescence microscopy, we used "INCA-DAPI" solution, which consists of 3-4 volumes of INCA buffer mixed with 1 volume of DAPI stock (10 μg/ml 4',6-diamidino-2-phenylindole (DAPI), 10 mg/ml p-phenylenediamine, 50% glycerol). This buffer must be made fresh and be protected from light.

To monitor cell breakage during grinding, aliquots for examination by fluorescence microscopy were periodically collected using a 200 μl pipettor fitted with tips that were bevel-clipped and bent slightly to form a scoop (Figure 1k). The stroke volume was set to 100 μl and the plunger held depressed to the first stop while the tip was used to scoop up a 4- to 6-mm-wide portion of the slurry of ground cells and liquid nitrogen from the rotating mortar bowl. Immediately after the liquid nitrogen evaporated from the aliquot (2-3 s) the pipette tip was plunged into 150-250 μl of "INCA-DAPI" mix in a 600-μl microcentrifuge tube. The plunger was then swiftly but smoothly released, drawing fluid through the ground cells to simultaneously thaw and disperse them. The pipetting cycle was quickly repeated 3-5 times to evenly mix the suspension before ~100 μl were diluted into a fresh 200-300 μl volume of ice-cold INCA-DAPI mix. Care must be taken to avoid generating bubbles or foam during the entire process. The remainder of the initial suspension was also stored on ice.

Pre-grinding samples were cut or chipped from frozen "noodles" using sterile razor or scalpel blades and/or forceps.

Slides for microscopy were loaded with 7-10 μl of direct or diluted suspension. Alternatively 5 μl of sample were mixed with 5 μl DAPI stock. Lower volumes often resulted in crushing of nuclei upon mounting the cover slip, while larger volumes increased interference by fluorescence from beyond the selected focal plane.

### Direct isolation of high-molecular-weight (HMW) DNA and replication intermediates (RIs) from cryo-ground cells

A homogeneous suspension of proteinase K (Roche #03115879001, recombinant, PCR grade) at 1.5 mg/ml was prepared in TEN buffer (50 mM Tris-HCl, pH 8.0, Invitrogen #15568-025, nuclease-free; 50 mM EDTA, pH 8.0, GIBCO # 15575-038, nuclease free; 100 mM NaCl, Ultrapure H_2_O GIBCO #10977-015, nuclease-free), then chilled on ice. Stored ground metabolically-fixed (azide-treated) log-phase 972 h- cells were transferred from the freezer and weighed in the same manner as described above for the noodles. Nine g of ground cells, corresponding to an 8-ml volume of packed cells (6.7 × 10^10^), were sprinkled in 1- to 2-g portions into 200 ml ice-cold Proteinase K/TEN suspension contained in a 2-l glass beaker. Each portion was allowed to thaw and disperse (with gentle agitation as needed) before the next was added. The storage tube holding ground cells was held in liquid nitrogen between transfers.

When all of the ground cells had been evenly dispersed into the proteinase K suspension (~18 min total for the 9 g), 10.9 ml of 30% (g/ml) aqueous Sarkosyl (sodium lauroyl sarcosinate, Sigma, #L5777) were added to the nominally-208-ml combined suspension volume to give a 1:19 dilution, resulting in a final concentration of 1.5%. The detergent was added by gentle swirling, and the mixture was carefully poured into a 500-ml polypropylene centrifuge bottle, which was then sealed and placed in a 37°C water bath for 3.5 h (hr), after which insoluble materials were pelleted by centrifugation for 5 min at 5,000 rpm, 2°C in a Sorvall SLA-3000 rotor. We used 37°C for our proteinase-K digest rather than the enzyme's optimum of 55°C-60°C in order to minimize loss of RIs due to branch migration and nascent-strand extrusion.

The clear supernatant was carefully decanted into a sterile, 250-ml polypropylene graduated cylinder and its volume recorded (212 ml in this example), then adjusted to 254 ml with 19:1 TEN:30% Sarkosyl. The supernatant was then poured into a fresh 500-ml polypropylene centrifuge bottle prior to the addition of 266.70 g CsCl (Sigma, #3032). The bottle was sealed and rolled gently to-and-fro until the CsCl was completely dissolved (30-40 min). A total solution volume of 320 ml was confirmed in a 500-ml graduated cylinder, and then 320 μl of 5-mg/ml aqueous Hoechst 33258 dye (Invitrogen, #H1398) were added. The solution was gently mixed by pouring into the 500-ml centrifuge bottle, which was then resealed and allowed to stand overnight at 4°C. A small amount of white suspended precipitate formed but cleared upon re-warming of the solution to room temperature. The CsCl solution was then distributed in 40-ml portions among Quick-Seal polyallomer ultracentrifuge tubes using a pasteur pipette cut and fused mid-barrel to a 50-ml conical disposable polypropylene centrifuge tube, as a funnel to allow a smooth, continuous flow (Additional File 2), which minimized mechanical shear. Isopycnic ultracentrifugation was performed in a Beckman VTi-50 rotor at 45,000 rpm and 20°C for 44-45 h.

### Collection and selective precipitation of purified HMW DNA

The banded Hoechst-stained chromosomal DNA was visualized by long-wave (360 nm) UV illumination (Additional File 3A), then carefully harvested by side puncture using 3-ml disposable syringes fitted with 18-gauge hypodermic needles (Additional File 3B), making sure that the needle punctures the tube on the side opposite the pellet of RNA, salt and debris. Behavior of the banded material as a single, tangled and/or viscous mass as it is drawn into the needle indicates high molecular weight and purity of the DNA. To reduce shear, the needle was removed from the syringe before each collected band was deposited into a pool. This pool (24-25 ml total) was gently mixed by inversion, then divided by wide-bore serological pipette into 2.4-ml aliquots, each in a 15-ml conical screw-cap polypropylene tube at room temperature. In our experience, this partitioning is necessary to avoid problems with re-dissolving and dispersion of the HMW DNA precipitate.

Selective precipitation of HMW DNA into discrete, singular (one per aliquot) "cocoons" (Additional File 3C) was initiated by layering 3 volumes (7.2 ml) of 23°C, 75% (v/v) reagent-grade ethanol (Pharmco-AAPER, #111ACS200 diluted in nuclease-free water (GIBCO #10977-015)) onto each aliquot, avoiding disturbance to the interfaces (it may be best to process each aliquot individually at this step). The appearance of white filamentous material at the interface, often extending upward into the alcohol, indicated the start of HMW DNA precipitation. The tube was then gently swirled and rocked to begin condensation of the precipitating HMW DNA into a single "cocoon" and to gradually mix the CsCl solution into the ethanol to complete the precipitation. Care must be taken at this stage to minimize separation of the growing DNA cocoon from the interface (Additional File 3C) to avoid shearing of longer molecules still partially dissolved in the remaining aqueous phase. The nebulous cocoon first expands rapidly during the mixing as the accumulation of precipitating HMW DNA peaks (Additional File 3C) but then condenses (Additional File 3D) as a result of increasing dehydration by the ethanol. Mixing was completed by gentle inversion. Once the cocoon was fully condensed and settled to the tube bottom or adhering to the wall (Additional File 3D), the ethanol mixture, occasionally clouded by precipitates of low-molecular-weight DNA, RNA and other contaminants, was thoroughly decanted and replaced with 10 ml of 23°C, 70% ethanol. The cocoon was rinsed by inversion of the sealed tube, then allowed to soak at room temperature for at least 2 h to extract residual salts (primarily CsCl) and other trace contaminants. The wash was decanted and replaced by another 10 ml of 70% (v/v) ethanol for storage at 4°C and shipment (in our case, from Buffalo, NY, to Charlottesville, VA).

### Two-dimensional gel analysis of DNA replication intermediates

The HMW DNA cocoons were drained of 70% EtOH, air-dried until only a barely detectable amount of moisture remained (complete drying renders subsequent re-hydration much more difficult), then resuspended in TEN (10 mM Tris-Cl, pH 8.0, 1 mM EDTA, 10 mM NaCl) and allowed to rehydrate overnight. Identification and characterization of replication intermediates in DNA was performed as described previously, beginning at the DNA digestion step and proceeding from there [13,14].

### Suspension and washing of nuclei from cryo-ground cells

Stored ground cells were transferred from the freezer and weighed as described above. For each g of ground cells (excluding the mass of any previously-added frozen buffer in the case of "cryo-mixes"), four ml of INCA buffer (4°C) were placed in a polypropylene beaker chosen to give maximum surface area at a liquid depth of 2-3 cm. The ground material or cryo-mix was then sprinkled evenly over the surface of the INCA buffer in increments of ~1 g, as described for the DNA replication sample in the preceding section. Upon homogeneous dispersion of the full amount of ground material, the suspension was gently poured into pre-chilled 13-ml polypropylene round-bottom snap-cap tubes (16 × 100 mm, Sarstedt #62.515.006) or, as dictated by volumes, 40-ml polypropylene Oakridge bottles. Note: the rotor adapter sleeves (Corning #8441) used for the 13-ml tubes were trimmed (in balanced pairs) to accommodate having the tube caps in place during centrifugation to prevent spilling and contamination. The centrifuge tubes or bottles were centrifuged horizontally at 0°C in an HB-6 rotor (Sorvall) for 2 min at 3000 rpm to pellet aggregated debris and residual whole cells. The speed was then increased to 5000 rpm for 3 min, and finally to 8000 rpm for 5 min to recover nuclei-bearing cell fragments and released nuclei. The resulting supernatants were opalescent to slightly turbid and were removed via serological pipette. The tri-layered pellets were gently resuspended in another 4 ml of INCA buffer (0°C) per g of ground cells (see the first sentence of this paragraph). This suspension was centrifuged at 0°C in an HB-6 rotor at 5,740 rpm for 2 min to pellet aggregated material and intact cells. The milk-like supernatant was transferred to fresh tubes, leaving beige-colored pellets, then centrifuged in an HB-6 rotor at 0°C and 8,500 rpm for 7 min to collect the washed crude nuclei. The cleared, slightly opalescent supernatant was removed by pipette. The pellet was immediately but gently resuspended in a total of 1 ml of 0°C INCA buffer per original g of ground material for assay by fluorescence/phase-contrast microscopy and for use in chromatin analysis.

### Micrococcal nuclease (MNase) digestion of DNA in permeabilized nuclei

The suspensions of washed, crude nuclei were pooled as appropriate, and the total volume was adjusted to 1 ml per 5 × 10^9 ^initial cell equivalents with 25°C INCA buffer. The pooled material was quickly equilibrated to 25°C in a water bath. After a gentle remixing of the pool by inversion, a 100-μl aliquot was transferred to a 1.5-ml conical polypropylene microcentrifuge tube and gently mixed by micro-pipettor with an equal volume of INCA-NP buffer (0.15% v/v NP-40 nonionic detergent [Pierce Surfact-Amps, product #28324] in INCA buffer) at 25°C. This mixture served as an "exogenous Enzyme-Free" (EF) control for the MNase digestion. The main portion of the suspension was then gently mixed with an equal volume of INCA-NP at 25°C containing 300 units/ml (U/ml) MNase (U.S. Biochemicals, #70196Y, stored at -20°C as 15 U/μl stock solution in 50% v/v sterile glycerol) in a 13 ml, 100 × 16 mm, round-bottom, snap-cap polypropylene tube.

The MNase reaction (150 U/ml) and "EF" control were incubated at 25°C with periodic mixing by gentle inversion for 12 min, then terminated by rapid mixing with 1/8 volume of STOP mix (0.25 M Na_2_-EDTA, 5% SDS, pre-heated to 65°C) followed by incubation at 65°C for 3-11 h to fully denature proteins and to reverse the formaldehyde cross-links. After a slow cooling to 55°C over an additional 2 h, 20 mg/ml Proteinase K was added to a final concentration of 500 μg/ml, and the incubation was continued for 10-18 h with occasional agitation.

### Purification of DNA after MNase digestion

Upon completion of the proteinase K treatment at 55°C, the samples were mixed by inversion, and the tubes were placed in wet ice for 30 min. The MNase reaction sample was then centrifuged at 8000 rpm, 2°C, for 20 min in an SS-34 rotor (Dupont-Sorvall) to pellet insoluble material, again using trimmed adapter sleeves to allow the tubes to remain capped. The smaller EF-control sample was centrifuged at 14,000 rpm, 1°C, in a Beckman-Coulter F24-1.5 rotor.

The clear supernatants were then extracted once with equal volumes of buffer-saturated phenol (pH 7.49-7.79 at 25°C, nuclease-free, Invitrogen #15513-039), then twice with equal volumes of chloroform:isoamyl alcohol (24:1; SIGMA #C-0549). NOTE: the aqueous phase was denser than the phenol phase due to the high sorbitol content of the INCA buffer. The volumes of the cleared final aqueous phases were measured, and ammonium acetate was added to give a concentration of 2.5 M (from a 10 M stock solution). Absolute ethanol (room temperature [~25°C]; Pharmco-AAPER #111ACS200) was added to a final concentration of 71.4% v/v (i.e. 2.5 × the combined volumes of the sample and the added ammonium acetate stock) and mixed in by inversion. Precipitation of DNA was allowed to proceed overnight at room temperature. Precipitated material was pelleted by centrifugation for 40 min at 8000 rpm, 20°C, in an SS-34 rotor in the case of the MNase-treated sample and at 14,000 rpm in a Beckman-Coulter F24-1.5 rotor for the EF-control sample. The supernatants were thoroughly removed, then replaced with equal volumes of fresh, room-temperature 70% v/v ethanol, and the pellets were soaked for 7-15 min to extract residual salts and organics. After this incubation, the tubes were re-centrifuged as above, but for only 5-10 min, to firmly re-seat the pellets, thus facilitating safe, thorough removal of the 70%-ethanol wash from the tubes. The pellets were then air-dried to just the point of transparency before addition of 0.5-1.0 ml per MNase-treated sample (~10^10 ^original cell equivalents), or 100-150 μl per EF-control sample, of RNaseI solution (37°C, 50 mM Tris-HCl, pH 7.5, 50 mM EDTA, 150 U/ml RNaseI, MBI-Fermentas #EN0601). Rehydration and resuspension were allowed to proceed passively at 37°C for 20-60 min before solution was actively encouraged via pipetting. Incubation at 37°C was then continued for 2-16 h until RNA removal was judged complete by agarose electrophoresis assay of small aliquots of the reactions. The samples were then made 2.5 M in ammonium acetate, heated to 65°C for 30 min to permanently denature the RNaseI, which was precipitated and removed along with other residual proteinaceous matter, by incubation on ice for 20-60 min followed by centrifugation for 20-30 min at 0°C in either an SS34 rotor at 8000 rpm or a Beckman-Coulter F24-1.5 rotor at 14000 rpm. The resulting supernatants were transferred to fresh tubes and the DNA precipitated with room-temperature ethanol and pelleted as before.

### Agarose-gel-electrophoretic size selection of mono-nucleosomal DNA

Cleaned, pelleted MNase-digested DNA was dissolved in 300 μl of TE (10 mM Tris-HCl, pH 8.0, 1 mM EDTA, pH 8.0 (nuclease-free, Invitrogen, #AM9858)) per 1 × 10^10^-4 × 10^10 ^original cell equivalents, and then quantitated using a NanoDrop-1000 UV spectrophotometer (Nano Drop Technologies, Wilmington DE, USA). Up to 55 μg of sample (170 μg/ml), mixed with 1/5 volume of gel-loading solution (0.5% g/ml orange G (sodium salt, Sigma O-7252), 0.25% g/ml xylene cyanol, 15% g/ml Ficoll (type 400, Sigma F-4375), all in TE, pH 8.0) were loaded into an 81-mm × 2-mm × 2.2-3.0-mm well of a 3.5- to 4-mm thick, 1.25% g/ml NuSieve GTG (Lonza #50081) low-melting-temperature-agarose gel in 0.5 × TBE, 0.2 μg/ml ethidium bromide, and electrophoresed between marker ladders (50-bp DNA ladder, Invitrogen; Quanti-ladder, OriGene) at 4 V/cm (electrode separation), 4°C for 4 h. These conditions optimized resolution of nucleosomal DNA bands with minimal diffusional spreading. The top surface of the gel was kept clear of the running buffer (0.5 × TBE, 0.2 μg/ml ethidium bromide) to avoid loss of the small mononucleosomal DNA fragments through leaching.

Gels were photographed using brief 360-nm UV transillumination to identify the positions of the desired DNA bands. UV exposure was kept to a minimum to avoid damage to the DNA, despite the lower risk at 360 nm versus 300 nm. Selected bands were excised using sterile stainless-steel razor blades and a small, ethanol-wiped spatula. The excised bands ("explants") were transferred to labeled, tared, sterile disposable 13-ml polypropylene snap-cap tubes, weighed, and then stored at 4°C until used.

### Enzymatic recovery of DNA from agarose gel explants

**Figure 2:**
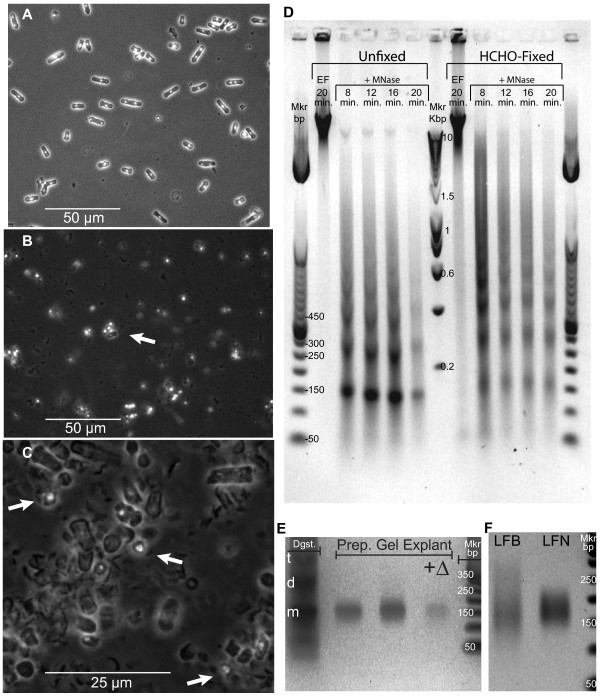
**Micrococcal nuclease (MNase) digestion of chromatin within fission yeast nuclei and recovery of protected DNA**. **(a**) DAPI fluorescence/phase contrast micrograph of log-phase *S. pombe *cells flash-frozen to -196°C then thawed in INCA buffer (see Materials and Methods). **(b) **Cryo-ground log-phase cells suspended and thawed in INCA buffer. The arrow marks an intact cell adjacent to a group of discrete liberated nuclei. **(c) **Oil-immersion micrograph of similarly-prepared stationary-phase nuclei. Arrows denote in-focus, liberated nuclei displaying the typical *S. pombe *tri-lobed DAPI fluorescence pattern. The ratio of liberated nuclei to cell debris seems lower here than in (B) because the microscope is focused on a thin (due to high magnification) plane containing settled cell walls, but many of the released nuclei are floating above that plane. **(d) **Gel-electrophoretic analysis of DNA recovered from MNase-digested nuclei of native ("Unfixed") and formaldehyde-treated ("HCHO-Fixed") stationary phase cells. "EF" indicates exogenous-Enzyme-Free control incubations. "+MNase" denotes incubation at 25°C with 150 units of MNase per ml for the indicated times. "Mkr bp" is a commercial 50-bp DNA-size-marker ladder. "Mkr Kbp" is a commercial 0.2- to 10-kbp DNA-size-marker ladder. **(e) **Gel-electrophoretic analysis of "mononucleosomal" DNA recovered from a band excised from a preparative-scale gel ("explant"). Twice as much DNA was loaded into the third and fourth lanes as into the second lane. "Dgst" indicates total DNA recovered from the parent MNase digest (12 min) of unfixed, stationary-phase nuclei; "t", "d" and "m" respectively mark the tri-, di-, and mononucleosomal DNA bands. "+Δ" signifies heat-denaturation of sample immediately prior to loading as an assay for internal nicking. (**f**) Gel-electrophoretic analysis of DNA recovered from a broad explant centered on the "mononucleosomal" band of a preparative-scale gel ("LFB": Log-phase, Fixed cells, Broad explant), then run on a second preparative gel from which the "mononucleosomal" band was re-sampled as a narrow explant ("LFN": Log, Fixed, Narrow).

Sample DNA was recovered from the excised bands by digestion of the agarose matrix using Gelase (Epicentre #G09100) essentially according to the manufacturer's "fast" protocol adjusted for 0.5 × TBE, but with the following modifications. The reaction time at 45°C was extended to 2 h at 15 U Gelase per g of excised band or to 12 h at 6.5 U per g excised band. After the 45°C incubation, the reaction was chilled on ice for 10-30 min, and its viscosity was assessed visually as a test for adequate digestion of agarose. Completed Gelase reactions were centrifuged in an SS-34 rotor at 10,000 rpm, 0°C, for 10-15 min to pellet residual insoluble material. The resulting supernatants were transferred to appropriately labeled, fresh tubes and made 2.5 M in ammonium acetate for removal of the Gelase and other salt-precipitable contaminants at 0°C as described for the RNaseI reaction in the preceding section, prior to room-temperature ethanol precipitation of the DNA. The pelleted DNA was re-dissolved in TE (pH 8.0) at 200 μl per original 1 × 10^10^-4 × 10^10 ^cell equivalents. Once dissolved, the samples were transferred to appropriately labeled 1.5-ml polypropylene conical microfuge tubes, then 1/9 volume of 3 M sodium acetate, pH 5.6, was mixed in, followed by the addition of room temperature absolute ethanol to a final concentration of 71% v/v. DNA re-precipitation was allowed to proceed at 4°C overnight followed by centrifugation at 14,000 rpm, 20°C in a Beckman-Coulter F24-1.5 rotor for 40 min. These final pellets were rinsed twice with one supernatant volume of 70% v/v ethanol at room temperature, then air dried to near-transparency before solution in TE, pH 8.0, 40 μl per original 1 × 10^10^-4 × 10^10 ^cell equivalents. The quantity and integrity of the recovered DNA were assessed by both NanoDrop spectroscopy and ethidium bromide/agarose-gel electrophoresis (Figure 2e). One half of the remainder of each sample, or 15 μl containing 450-550 ng DNA, was transferred to a fresh, appropriately labeled microfuge tube and stored at or below 4°C pending sequencing.

### Analysis of nucleosomal DNA

Sequencing and analysis of mono-nucleosomal DNA were carried out by methods that are described in detail in a submitted manuscript (RM Givens, WKM Lai, JM Rizzo, JE Bard, PA Mieczkowski, J Leatherwood, JA Huberman and MJ Buck, "Chromatin architectures at fission yeast transcriptional promoters and replication origins"; henceforth referred to as "Givens et al., submitted for publication"). Here we provide a brief summary. DNA was sequenced by an Illumina Genome Analyzer IIx. Sequencing reads were aligned to the August, 2008, build of the *S. pombe *genome ftp://ftp.sanger.ac.uk//pub2/yeast/pombe/Chromosome_contigs/OLD/20080827 using the BowTie algorithm ([15]; http://bowtie-bio.sourceforge.net/index.shtml). MNase protection was calculated for the dataset of aligned sequence reads by extrapolating each read to a final length of 120 nucleotides, then adding up the number of extended reads crossing each position in the genome, then dividing the number of reads at each position by the genome-wide average read count per bp. The 120 nt value for sequence tag extrapolation length was empirically selected as yielding the highest resolution of nucleosomes in genome-wide average profiles of transcription units aligned by start site.

## Results and discussion

### Cryo-fixation and precision grinding of fission yeast in liquid nitrogen

Figures 2a-c show, by combined phase-contrast and DAPI-fluorescence microscopy, the structures of flash-frozen, log-phase fission yeast cells and of their nuclei as released by our protocol. Prior to grinding, each log-phase cell has one or two brightly stained nuclei (Figure 2a).

For comparison with log-phase cells, we used phase-contrast microscopy to examine cells that were in stationary phase. Additional File 1A, B shows that stationary-phase cells are more uniform in morphology and generally shorter than log-phase cells. In addition, formaldehyde-fixed stationary phase cells (Additional File 1B) appear brighter by phase contrast than unfixed cells (Additional File 1A).

After grinding (Figure 2b), many of the nuclei appear diffuse because they are free to occupy positions beyond a single focal plane (in contrast to nuclei constrained within fission yeast cells of fairly uniform diameter, which tend to settle onto the slide surface as in Figure 2a). The arrow in Figure 2b points to a cluster containing several in-focus released nuclei and one still-intact cell for comparison. The fluorescence-photomicrograph in Figure 2c was taken at a higher magnification (oil immersion). The arrows in Figure 2c point to released, in-focus nuclei displaying the horseshoe-like, tri-lobed structure characteristic of nuclei within living fission yeast cells [16]. Thus our procedure enables simple bulk harvesting of fission yeast nuclei that are morphologically intact at the resolution of oil-immersion, phase-contrast, DAPI-fluorescence microscopy.

### Integrity of chromosomal DNA in released nuclei

Endogenous nuclease activity is frequently a problem with other methods for breaking fission yeast cells ([2,17]; RMG and JAH, unpublished observations). When nuclei released and washed using our protocol were incubated in INCA buffer supplemented with non-ionic detergent (NP-40) for 20 min in the absence of MNase, their DNA remained essentially intact, at double-strand sizes larger than 10 kb (Figure 2d, "EF" lanes). This indicates that our method yields fission yeast nuclei that are free of detectable detergent-stimulated endogenous nuclease activity.

### Effects of *in vivo *crosslinking by 1.5%-formaldehyde on kinetics of MNase digestion of fission yeast chromatin

In Figure 2d, we show the results of experiments comparing the MNase digestion kinetics of chromatin from formaldehyde-fixed ("HCHO-Fixed") cells to parallel native material ("Unfixed"). For unfixed chromatin, there is an evident increase in the proportion of monomeric nucleosomes (at about 150 bp) over the time course of 8-20 min. However, for fixed chromatin, a significant portion of tetra-, tri-, and di-nucleosomes are only very slowly, if at all, cleaved into monomeric nucleosomes, similar to results for fixed fission yeast chromatin obtained by Lantermann et al. (Figure 1c, right side [7]). This behavior of the fixed material persists at MNase concentrations at least 50% higher coupled with incubation times at least 50% longer than used in Figure 2d (Additional File 4), suggesting that, in these particular oligonucleosomes, formaldehyde crosslinks reduce the MNase sensitivity of the linker DNA to a level similar to that of DNA bound to the histone octamer. This effect of formaldehyde crosslinking would impose a two-part sampling bias against any class of nucleosomes having an above-average tendency to form these resistant linkers: exclusion by size selection in the case of partial digests and loss by internal cleavage in digests that leave only mononucleosomes. Thus, while physically preserving position information for most nucleosomes, formaldehyde crosslinking may ironically result in relative loss of signal from certain populations involved in higher-order structure.

For our analytical experiments we chose a moderate MNase incubation time (12 min) that provided good yields of mono-nucleosomes while retaining intermediate products up to at least tetra-nucleosomes (Figure 2d), thus presumably minimizing selective loss of signal from any nucleosomes exceptionally prone to internal digestion. After DNA purification, mono-nucleosome-sized DNA was collected by preparative agarose gel electrophoresis. Figure 2e demonstrates that DNA fragments recovered from the mono-nucleosome band in the preparative gel ("Prep. Gel Explant") have the same size range as the mono-nucleosome band ("m") direct from the MNase digest ("Dgst.") lane. The single, discrete band visible at a similar position in the lane containing the heat-denatured sample ("+Δ"), confirms the general absence of internal nicks (the weaker signal is due primarily to the poor fluorescence of ethidium bromide bound to single-stranded DNA).

We also noted that, in addition to a denser general smear, the MNase digestion ladders from formaldehyde-fixed samples exhibit broader, more diffuse bands in comparison to parallel unfixed material (Figure 2d). This led us to investigate the effects of the range of DNA fragment sizes encompassed by the prep-gel explants on the clarity and nature of the final results (Figure 2f and Additional File 1C). The DNA comprising the "Log Fixed Broad" sample (LFB) was recovered from a broadly cut prep-gel explant that included fragments ranging from ~100 bp to ~300 bp. A portion of the purified LFB DNA was resolved on a second prep gel, from which only the visually distinct mono-nucleosome band (running between ~150 and ~220 bp) was collected. We called this subset "Log Fixed Narrow" (LFN; Figure 2f and Additional File 1C). We provide detailed comparisons of the LFB and LFN samples in a manuscript (Givens et al., submitted for publication), where we show that the LFB signal is enriched relative to the LFN signal within the nucleosome-poor regions associated with transcriptional promoters and with DNA replication origins.

### Cryo-grinding and INCA buffer maintain nucleosome positions in the fission yeast *ade6 *gene region

**Figure 3:**
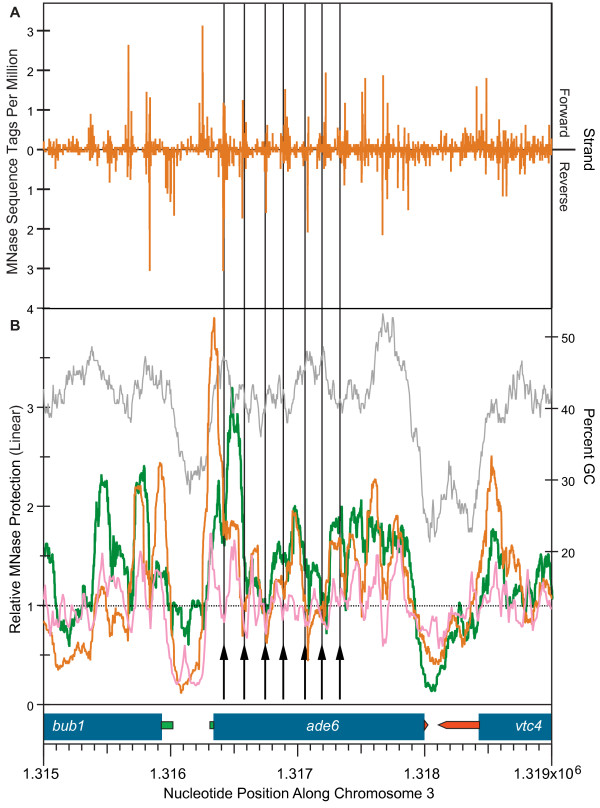
**Method-independent consistency of MNase cut sites and nucleosome positions within the fission yeast ade6 gene region**. The horizontal axis and transcription diagram at the bottom of this figure are common to panels **A **and **B**. Thick blue bars represent the CoDing Sequences (CDS) of the indicated genes. The narrow green bars represent the 5' untranslated regions (5'-UTRs) of these genes, while the narrow red pointed bars represent their 3'-UTRs. **(a) **Histogram (vertical orange bars) displaying the frequencies (tags at the indicated position per million tags genome-wide) of 36-nt sequence tags in mononucleosomal DNA (LFN sample; Figure 2f and Additional File 1C), which map uniquely to the forward (upward scale) and reverse (downward scale) strands of a 4-kbp region of *S. pombe *chromosome 3 centered on the *ade6 *gene. **(b) **Profiles of relative MNase protection. For the orange (LFN sample) and green (LUN sample) lines, this is defined as the frequency, relative to the genome average (horizontal black, dotted line), with which the indicated nucleotide position is crossed by computer-generated 3'-extrapolations of forward- and reverse-strand sequence tags to 120 bp. The pink line shows relative MNase protection based on the microarray results published by Lantermann *et al. *[21]. The percent GC (150-bp window) over the region is indicated by the gray line and right-hand vertical axis. The vertical black arrows and lines mark the positions of MNase cuts mapped within the *ade6 *gene by Lantermann *et al. *[7] using the traditional indirect-end-labeling method

The locations of nucleosomes in the *ade6 *gene region of fission yeast chromosome 3 have previously been measured, both by gel-electrophoretic analysis of MNase-generated fragment sizes (indirect end labeling; [7,18,19]), and, more recently, by micro-array analysis [20,21]. These previous measurements of nucleosome positions in the *ade6 *region provide a convenient standard for determining the reliability of data generated by any new approach, such as ours. In Figure 3a, note that the coding region of *ade6 *(see the gene diagram at the bottom of the figure) is marked by distinct spikes in tag frequency on the forward and reverse strands at regular intervals of approximately 130-170 bp, consistent with the presence of nine positioned nucleosomes. The vertical black lines that extend up into panel **A **from the arrows in panel **B **show the positions of MNase cut sites previously determined by gel electrophoresis and indirect end labeling [7].

The orange line in Figure 3b shows the "relative MNase protection" profile across this region for our LFN (Log-phase, Formaldehyde-fixed, Narrow band cut) sample, while the green line shows the same information for our LUN (Log-phase, Unfixed, Narrow band cut) sample (the other two samples [Additional File 1C] provided similar results with regard to nucleosome positioning; Givens et al., submitted for publication). The orange profile in Figure 3b was calculated from the raw data in Figure 3a, as explained in Methods and in the figure legend, while the green profile in Figure 3b was similarly calculated from the different raw data set obtained for our LUN sample (Givens et al., submitted for publication). For comparison, the pink line in Figure 3b shows the signal for relative MNase protection obtained by Lantermann et al. [21]. These investigators digested formaldehyde-fixed chromatin from log-phase fission yeast cells with MNase, isolated mononucleosomes, and then hybridized the mononucleosome DNA to a high-resolution tiled microarray [21].

For all of the MNase-protection profiles shown in Figure 3b, the major peaks suggest locations of nucleosomes. The heights of these peaks provide a measure similar to relative "nucleosome occupancy" [22], but we prefer relative "MNase protection", since resistance to digestion may be conferred or modulated by other nucleoprotein complexes or by nucleotide sequence [23,24]. It is striking that, in regions where the nucleosomes appear to be strongly positioned (strong periodic signals in Figure 3a; the 3' end of the *bub1 *gene and the entire *ade6 *gene), there is excellent agreement with regard to nucleosome positions (though not signal strengths) among the three methods for determining nucleosome positions and among the various preparation methods: cryo-grinding (orange and green lines) versus spheroplasting (pink line and black vertical lines), and formaldehyde-fixed cells (orange and pink lines) versus unfixed cells (green line and black vertical lines). The identity of nucleosome positions between formaldehyde-fixed and unfixed cells (Figure 3b) argues that, when either our cryo-grinding method or the spheroplasting method employed by Lantermann et al. [7] for indirect end labeling is employed to prepare nuclei or chromatin from unfixed cells for subsequent MNase digestion, no sliding of nucleosomes takes place during the MNase digestion. When chromatin is prepared from unfixed fission yeast cells by a variety of other spheroplasting methods, as well as by grinding with a Waring blender under liquid nitrogen, nucleosomes frequently slide together during subsequent incubation with exogenous MNase, giving rise to artifactually short inter-nucleosome-repeat distances (see reference [1] for a review of nucleosome sliding as well as for a list of preparation methods that permit nucleosome sliding).

Overall, Figure 3 demonstrates that our raw data and our processed results are consistent for the *ade6 *region with the indirect-end-labeling data of Lantermann et al. (Figure 3b, black arrows and vertical lines [7]) and with the nucleosome positions determined by genome-wide hybridization to microarrays (Figure 3b, pink line [7,21]). Thus our new analyses confirm and extend previous results [7,21] indicating that many (but not all) nucleosome positions in fission yeast are robustly conserved despite significant variations in methods of nuclear preparation and nucleosome localization.

### Integrity of DNA replication intermediates in nuclei released by cryo-grinding

**Figure 4:**
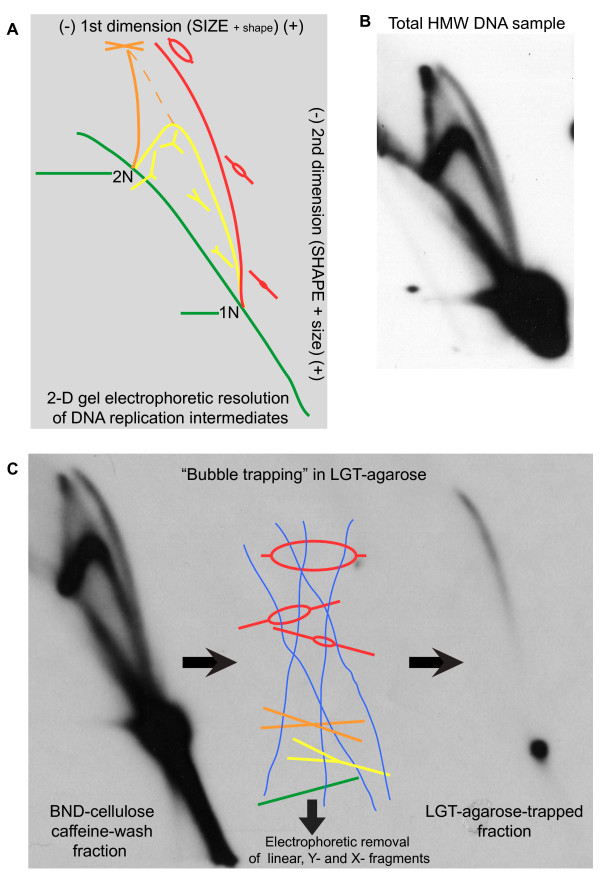
**Integrity of DNA replication intermediates (RIs) in fission yeast cryo-ground cells**. **(a) **Schematic depiction (based on the pioneering study by Brewer and Fangman [25]) of Southern blot signals from two-dimensional (2D) gel-electrophoretic resolution of a restriction-endonuclease-digested, asynchronously-replicating DNA population. Axis labels indicate the primary resolution criteria in each electrophoretic direction, with font size conveying relative influence of given factors. Color-matched cartoon renderings depict the comparative structures and sizes of probe-targeted fragments responsible for specific features of the signal pattern. Green: linear fragments ("arc of linears"), 1N: migration position of single-length simple linear version of probe-targeted fragment; 2N: migration position of double-length, simple linear version of probe-targeted fragment; Yellow: fragments bearing single, externally-initiated replication forks ("Y arc"); Orange: fragments bearing two converging, externally initiated replication forks ("X line"); Red: fragments bearing diverging replication forks, initiated from a common internally located origin ("Bubble Arc"). **(b) **Autoradiogram from 2D analysis of the 3-kb fission yeast rDNA *Kpn*I/*Hin*dIII digestion fragment using HMW DNA prepared from our 972 h-wild-type cryo-ground cells. **(c) **Left: Autoradiogram from 2D analysis of DNA from panel **B **after enrichment for RIs by Benzoylated Naphthoylated DEAE-cellulose chromatography. Center: Schematic representation of selective retention of bubble-bearing DNA fragments in Low-Gelling-Temperature (LGT) agarose (blue filaments) by topological capture during gel polymerization. Right: Autoradiograph from 2D analysis of topologically purified bubble-bearing fragments.

Replicating DNA structures are among the most transient and labile in the cell. Figure 4 demonstrates that cryo-grinding permits convenient milligram-scale isolation of high-molecular-weight (HMW) fission yeast DNA bearing abundant, intact replication intermediates (RIs). Panel **A **is a schematic depiction of the patterns generated by the various RIs of a given restriction fragment during two-dimensional (2D) gel electrophoresis, as first described by Brewer and Fangman [25]. Panel **B **presents the results of such an analysis on a sample of fission yeast HMW DNA prepared directly from log-phase fission yeast 972h- cryo-ground cells as described in Materials and Methods. The fragment probed in panel **B **is centered on *ars3001*, a locus within the ribosomal DNA repeat unit that is known to be an active origin of bi-directional replication *in vivo *[17,26]. Since any one copy of *ars3001 *initiates replication in only a minority of cell cycles, most of the fragments contain either single or converging replication forks. The autoradiogram clearly reveals that all classes of RIs are represented in the sample, including a good proportion of internally-initiated, divergent-fork- or "bubble"-bearing fragments, the most fragile type. The high proportion of bubble-containing fragments in Figure 4b should be compared with the *much *lower proportion obtained for *ars3001 *using a conventional bead-beating method for DNA isolation (Figures 3b, c in [17]).

Figure 4c illustrates the structural validation and purification of the active-origin fragments by agarose "bubble-trapping" [14], in which strands of polymerizing low-melting-temperature agarose extend through the ring-like portion of these RIs, thus mechanically retaining them while the remaining fragment types are electrophoretically removed (Figure 4c, center). The purified bubble fragments are recovered by re-melting the agarose, then analyzed by 2D gel electrophoresis. The left side of panel C shows a 2D gel pattern for DNA from the cryo-ground sample after enrichment for partially single-stranded fragments (such as RIs) by chromatography on benzoylated naphthoylated DEAE-cellulose (BND-cellulose; notice the increased ratio of forked structures to linear structures in this post-BND-cellulose sample compared to the pre-BND-cellulose sample in panel **B)**. The right side of panel **C **shows the 2D pattern of the trapped fraction. It consists almost entirely of material that migrates along a "bubble arc" (the red arc in Figure 4a). Thermal-induced extrusion of shorter nascent strands (branch migration) accounts for the decreased signal in the "early" (lower) portion of the "bubble" arc and for the small (relative to the starting material on the left side) amount of linear DNA at the 1N position (these are the parental strands remaining after nascent-strand extrusion). This bubble-trapped material is currently being analyzed to help identify and quantitate the frequency of usage of replication origins throughout the fission yeast genome (LD Mesner, RM Givens, JL Hamlin and JA Huberman, manuscript in preparation).

The yield of high-molecular-weight DNA in the preparation that gave rise to Figure 4 was 48% of theoretical (based on assuming 1.4 × 10^7 ^bp/haploid genome, 625 g/mol bp, and 80% G2 cells in mid-log phase). Since some DNA would have been lost during the DNA purification procedure, it seems likely that, in this case at least, the proportion of nuclei recovered was at least 50% of the nuclei in the starting cell population.

## Conclusions

Here we have described a simple method by which large quantities of cultured fission yeast cells can be literally frozen in their current metabolic and growth states (by flash-freezing in liquid nitrogen; "cryo-fixation"), then "cryo-ground" in a large, motor-driven, mortar-and-pestle apparatus at reproducible settings, leading to a high yield of partially purified, morphologically intact nuclei, as well as other cellular components. We have not examined the potential uses of the other cellular components. Presumably, with appropriate separation techniques, fractions enriched for any of the constituents of the cell that might be of interest to the investigator could be obtained.

We found that the partially purified nuclei from this procedure were suitable for chromatin structural studies. There was no degradation of nuclear DNA by endogenous nucleases, in contrast to some previous methods for chromatin analysis in fission yeast, which were plagued by such degradation (reviewed in [2]). Furthermore, even in the absence of formaldehyde fixation, there was no sliding of nucleosomes, which was a problem with several previous methods for fission yeast chromatin preparation (reviewed in [1]). We also determined that the partially purified nuclei are a good source of high-molecular-weight DNA, suitable for examination of replication, repair or recombination intermediates. Our yields of *ars3001 *replication intermediates were higher than previously obtained (using different nuclear isolation and DNA purification methods) from unsynchronized fission yeast cells [17,27] or even from cells synchronized to early S phase [28].

In summary, the simple, economical, reproducible method described here reliably yields partially purified fission yeast nuclei that are a superior starting material for a variety of experiments. In addition, with appropriate species-specific modifications, this method seems likely to prove useful for preparation of high-quality nuclei from the cells of other organisms with (or without) tough cell walls.

## Availability of supporting data

All of our raw sequencing data are available in the NCBI GEO database, accession number GSE28071. Our data are also available at http://www.acsu.buffalo.edu/~mjbuck/Fission_Yeast_chromatin.html. In addition to raw data at this web site, processed data, including relative MNase-protection profiles and template-filtered nucleosome positions for each of the three chromosomes are also provided.

## Competing interests

The authors declare that they have no competing interests.

## Authors' contributions

RMG developed the fission yeast cryo-grinding and nuclear isolation method, carried out the cryo-grinding, nuclear enrichment, DNA purification, and MNase digestion experiments, and wrote the first draft of the manuscript. LDM and JLH developed the bubble-trapping method. LDM carried out the 2D gel analyses. MJB provided intellectual guidance for the chromatin analysis experiments, and he processed the raw sequencing data to generate usable information regarding nucleosome locations. JAH wrote the second draft of the manuscript. All authors contributed to aspects of experimental design, participated in writing the manuscript, and approved the final version.

## Supplementary Material

Additional file 1 **Effect of fixation on stationary cell morphology, and pedigrees of experimental samples**. A stationary-phase *S. pombe *culture was sampled either directly **(A) **or after formaldehyde fixation **(B)**. These samples were then diluted in water and loaded onto a haemocytometer slide for counting and morphological evaluation. Fields were photographed at the same focal plane (note the resolution at the edges of the counting grid etchings) using the same microscope and camera settings. The labeled bar in the lower left of each panel shows a scale of 50 μm, which is also the size of the sides of the etched squares. **(c) **Pedigree of experimental samples. The samples were named according to the following conventions. "**L**" indicates that the cells were growing **L**ogarithmically when harvested (Figure 2a). "**S**" means that the cells were in **S**tationary phase when harvested (Panels **A **and **B**). "**F**" means that the cells were **F**ixed with formaldehyde prior to harvesting, while "**U**" indicates that the cells were **U**nfixed when harvested. "**B**" indicates that the band excised from the prep gel was **B**roader than usual and thus contained a wider range of fragment sizes (example in Figure 2f), while "**N**" means the excised band was relatively **N**arrow, with the intention of analyzing primarily fragments close to mono-nucleosome size (see Figure 2f for a comparison of size ranges). The cells used in the LUN, LFB and LFN samples came from the same batch of log-phase cells, while the cells used in the SUN sample came from an independent stationary-phase culture. For the LUN and SUN samples, the cells were not fixed with formaldehyde. See the main text for additional details. Click here for file

Additional file 2 **Custom funnel for loading heat-sealable ultracentrifuge tubes**. **(A) **Pasteur pipette cut mid-barrel, removing the upper constriction. **(B) **The tip (not shown) is flame polished to remove shear-generating edges, as is the rim of the cut barrel (shown), which is additionally given a slight exterior lip. The pipette is pressed (thin arrow) from the inside through a snug-fitting hole bored into the bottom of a 50-ml polypropylene conical screw-cap centrifuge tube until the lip is firmly seated. (thick arrow) The seal and stability of the assembly can be optionally promoted by carefully flame-heating the pipette barrel near the contact point, then gently pulling the lip slightly into the softening plastic. **(C) **Assembled funnel. The flame-polished tip of the pasteur pipette is kept just beneath the surface of the liquid as the polyallomer centrifuge tube fills. Note: the screw cap of the 50-ml polypropylene centrifuge tube can be used to regulate the flow rate, by regulating the flow of air into the funnel, once the funnel is filled. Click here for file

Additional file 3 **Collection and selective precipitation of HMW DNA from a CsCl gradient**. **(A) **After centrifugation, a centrifuge tube containing DNA and Hoechst 33258 dye in a CsCl gradient was photographed under 360 nm illumination, which induces light blue fluorescence when the dye is bound to DNA. The DNA is visible as a thick, blue band, floating at its isopycnic density in the centrifugation-induced CsCl density gradient. **(B) **An 18-gauge hypodermic needle, attached to a clamped 3-ml syringe, was inserted through the wall of the centrifuge tube until its tip was located within the DNA band. The syringe plunger was then withdrawn very slowly, so as to prevent shear and to permit the long DNA molecules that initially entered the needle to drag other DNA molecules behind them (as a consequence of their high viscosity). **(C) **Selective precipitation of very high molecular weight (vHMW) DNA fibers by condensation into a single "cocoon" near the interface between 75% ethanol and CsCl solution. The 75% ethanol was layered onto pooled CsCl gradient DNA bands (see Methods). The hazy cloud extending up from the interface and partially obscuring the forming cocoon is a precipitate of salts, low-molecular-weight DNA fragments, RNA and other contaminants that will be decanted upon completion of vHMW DNA precipitation. Any remainder will be removed by a 70% EtOH wash. **(D) **Examples of fully condensed cocoons in 70% ethanol. Click here for file

Additional file 4 **Gel-electrophoretic analysis of DNA from MNase-digested nuclei of unfixed and fixed stationary-phase fission yeast cells**. Half of the cells in a stationary-phase culture were fixed with formaldehyde, then both portions of the culture were cryo-fixed and ground to liberate nuclei. Washed nuclei were incubated in parallel at 25°C for the indicated times in INCA buffer, at the indicated MNase concentrations. The percentage labels denote the portion of the respective digest sample loaded into a given lane. "Mkr bp": 50-bp DNA-size-marker ladder. "Mkr Kbp": 0.2- to 10-kbp DNA-size-marker ladder. Click here for file
