# Supplementary material for: Integrity of chromatin and replicating DNA in nuclei released from fission yeast by semi-automated grinding in liquid nitrogen
Source: BMC Res Notes. 2011 Nov 16;4:499. doi: 10.1186/1756-0500-4-499 (PMC3235078; doi:10.1186/1756-0500-4-499)
Supplement: Additional file 1 — Effect of fixation on stationary cell morphology, and pedigrees of experimental samples. A stationary-phase S. pombe culture was sampled either directly (A) or after formaldehyde fixation (B). These samples were then diluted in water and loaded onto a haemocytometer slide for counting and morphological evaluation. Fields were photographed at the same focal plane (note the resolution at the edges of the counting grid etchings) using the same microscope and camera settings. The labeled bar in the lower left of each panel shows a scale of 50 μm, which is also the size of the sides of the etched squares. (c) Pedigree of experimental samples. The samples were named according to the following conventions. "L" indicates that the cells were growing Logarithmically when harvested (Figure 2a). "S" means that the cells were in Stationary phase when harvested (Panels A and B). "F" means that the cells were Fixed with formaldehyde prior to harvesting, while "U" indicates that the cells were Unfixed when harvested. "B" indicates that the band excised from the prep gel was Broader than usual and thus contained a wider range of fragment sizes (example in Figure 2f), while "N" means the excised band was relatively Narrow, with the intention of analyzing primarily fragments close to mono-nucleosome size (see Figure 2f for a comparison of size ranges). The cells used in the LUN, LFB and LFN samples came from the same batch of log-phase cells, while the cells used in the SUN sample came from an independent stationary-phase culture. For the LUN and SUN samples, the cells were not fixed with formaldehyde. See the main text for additional details. [file 1756-0500-4-499-S1.PDF]

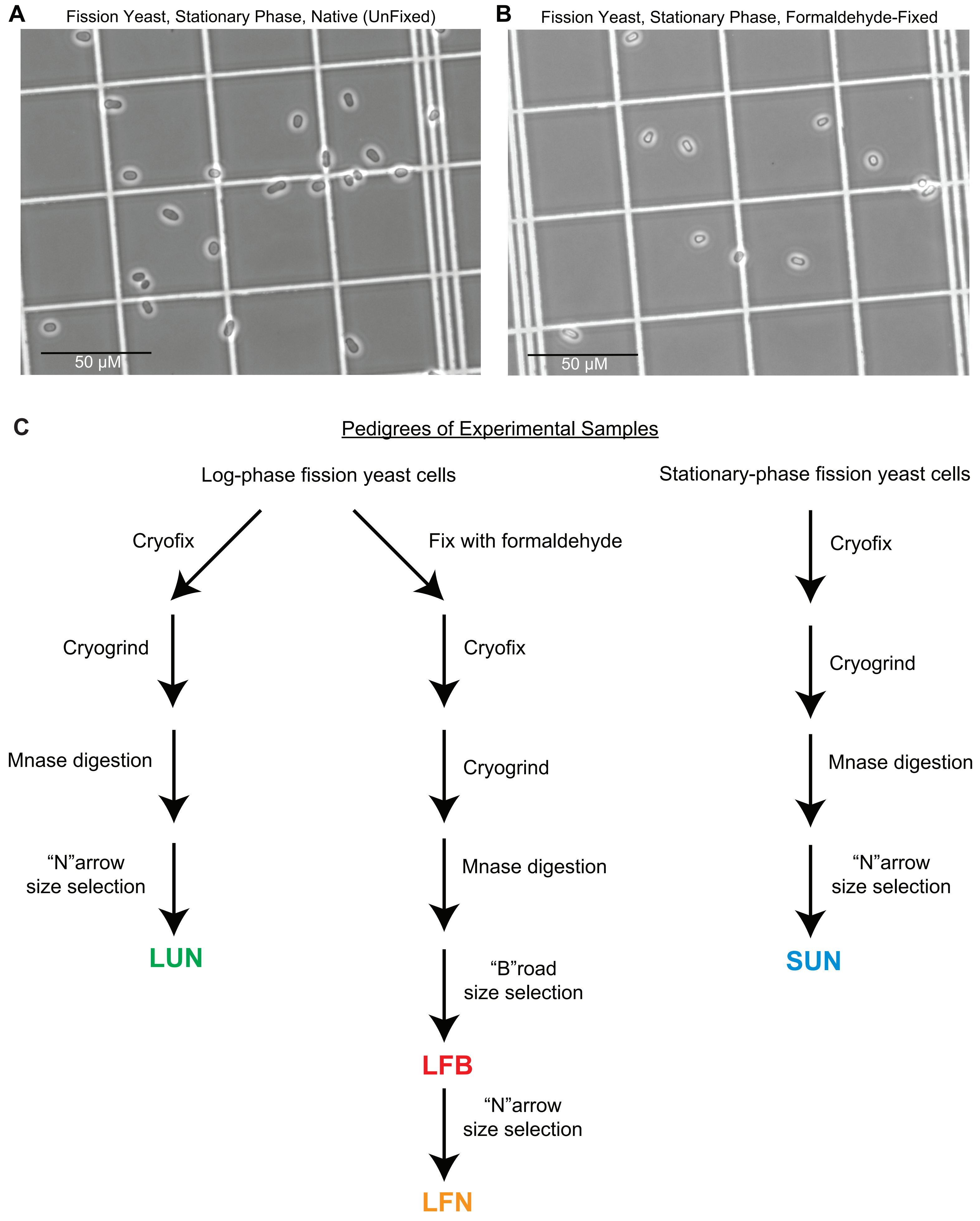
A
Fission Yeast, Stationary Phase, Native (UnFixed)
50 μM
B
Fission Yeast, Stationary Phase, Formaldehyde-Fixed
50 μM
C
Pedigrees of Experimental Samples
Log-phase fission yeast cells
Cryofix
Fix with formaldehyde
Cryogrind
Cryofix
Mnase digestion
Cryogrind
"N"arrow size selection
Mnase digestion
LUN
"B"road size selection
LFB
"N"arrow size selection
LFN
Stationary-phase fission yeast cells
Cryofix
Cryogrind
Mnase digestion
"N"arrow size selection
SUN
