# Supplementary figures and images for: Integrity of chromatin and replicating DNA in nuclei released from fission yeast by semi-automated grinding in liquid nitrogen
Source: BMC Res Notes. 2011 Nov 16;4:499. doi: 10.1186/1756-0500-4-499 (PMC3235078; doi:10.1186/1756-0500-4-499)

**A**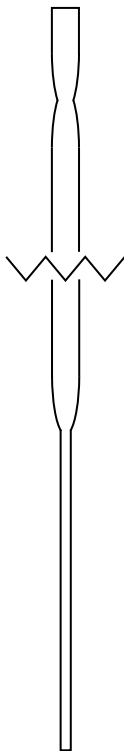**B**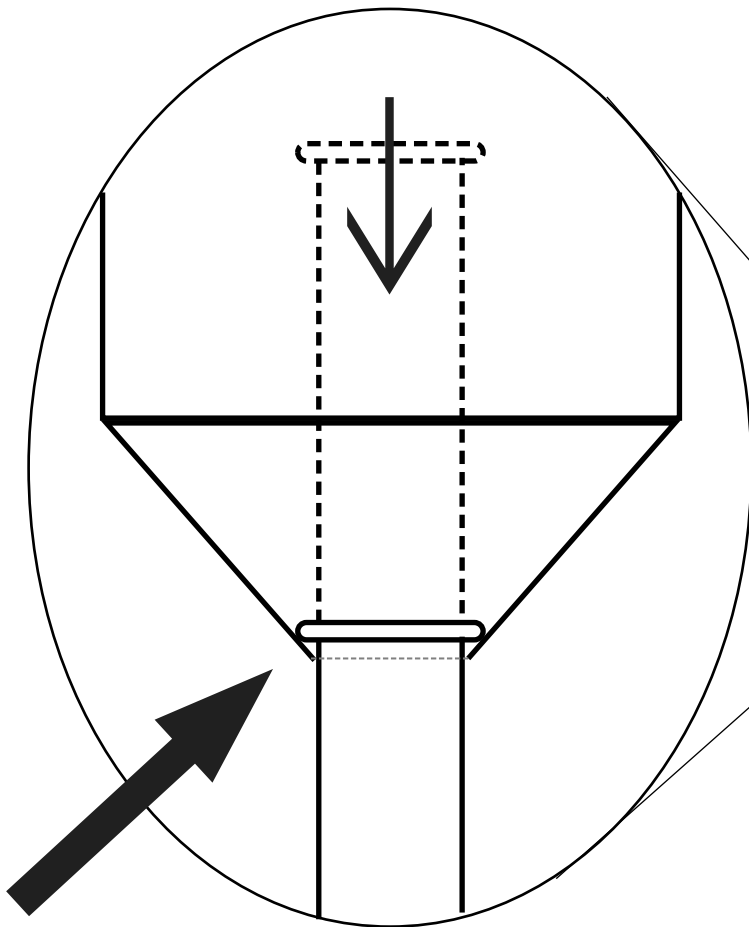**C**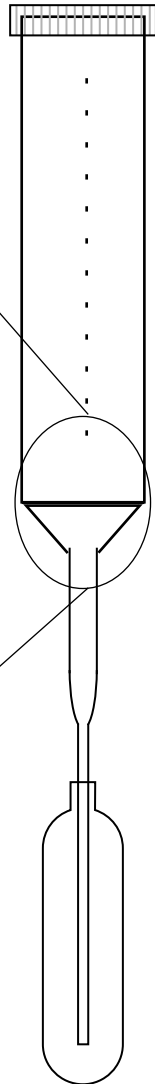

Supplement: Additional file 2 — Custom funnel for loading heat-sealable ultracentrifuge tubes. (A) Pasteur pipette cut mid-barrel, removing the upper constriction. (B) The tip (not shown) is flame polished to remove shear-generating edges, as is the rim of the cut barrel (shown), which is additionally given a slight exterior lip. The pipette is pressed (thin arrow) from the inside through a snug-fitting hole bored into the bottom of a 50-ml polypropylene conical screw-cap centrifuge tube until the lip is firmly seated. (thick arrow) The seal and stability of the assembly can be optionally promoted by carefully flame-heating the pipette barrel near the contact point, then gently pulling the lip slightly into the softening plastic. (C) Assembled funnel. The flame-polished tip of the pasteur pipette is kept just beneath the surface of the liquid as the polyallomer centrifuge tube fills. Note: the screw cap of the 50-ml polypropylene centrifuge tube can be used to regulate the flow rate, by regulating the flow of air into the funnel, once the funnel is filled. [file 1756-0500-4-499-S2.PDF]

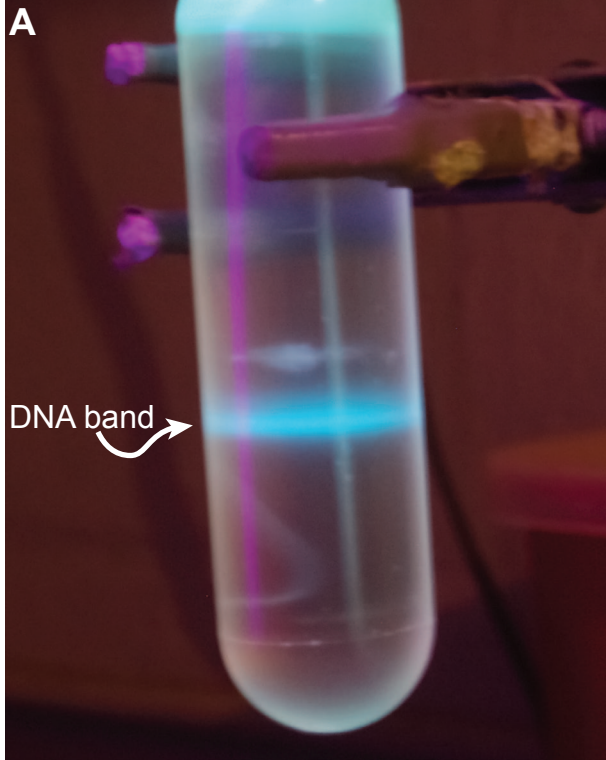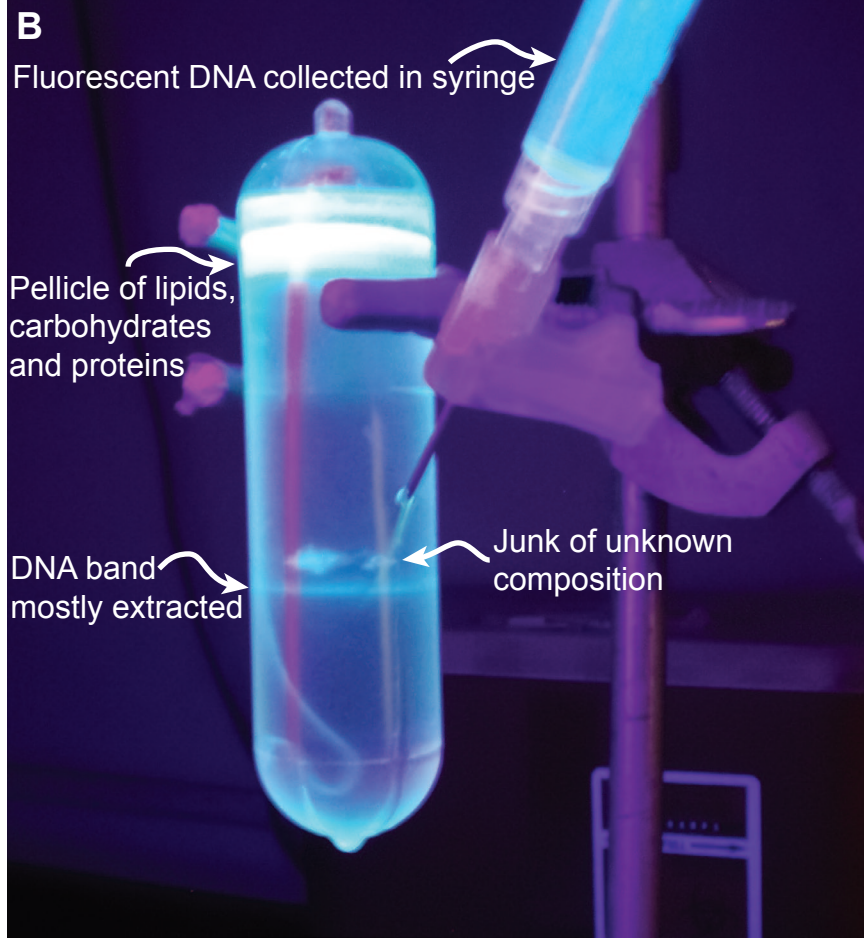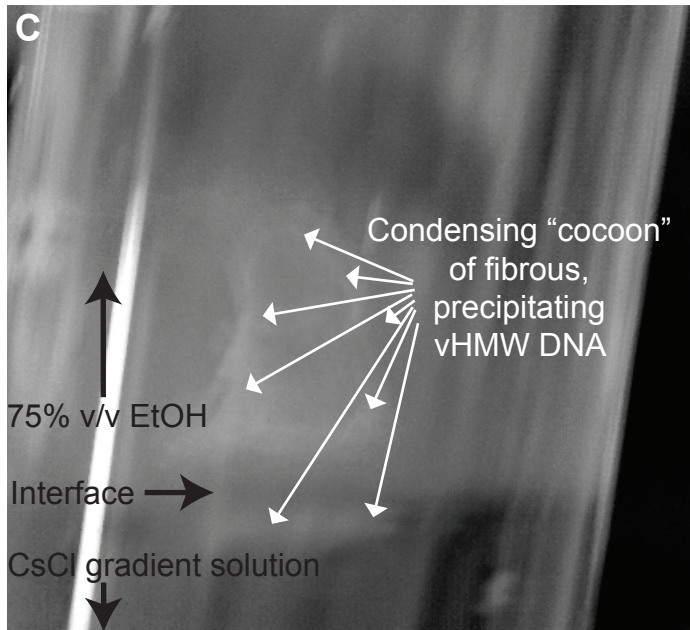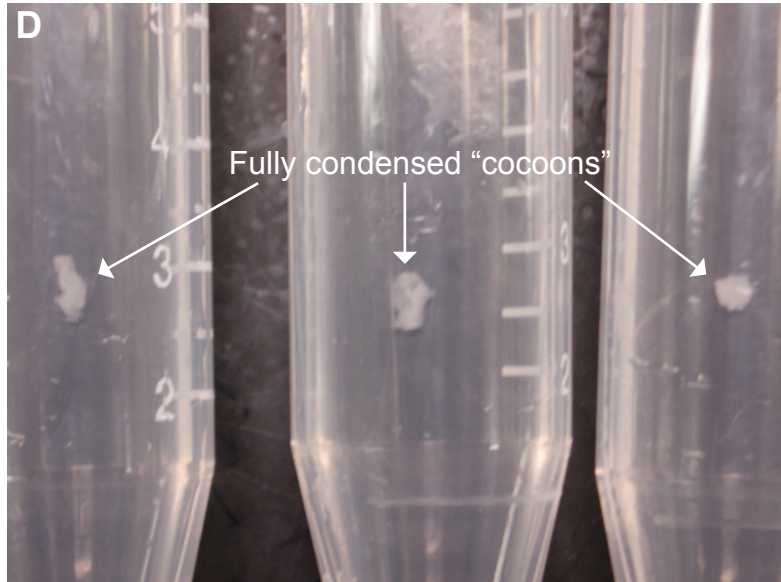

Supplement: Additional file 3 — Collection and selective precipitation of HMW DNA from a CsCl gradient. (A) After centrifugation, a centrifuge tube containing DNA and Hoechst 33258 dye in a CsCl gradient was photographed under 360 nm illumination, which induces light blue fluorescence when the dye is bound to DNA. The DNA is visible as a thick, blue band, floating at its isopycnic density in the centrifugation-induced CsCl density gradient. (B) An 18-gauge hypodermic needle, attached to a clamped 3-ml syringe, was inserted through the wall of the centrifuge tube until its tip was located within the DNA band. The syringe plunger was then withdrawn very slowly, so as to prevent shear and to permit the long DNA molecules that initially entered the needle to drag other DNA molecules behind them (as a consequence of their high viscosity). (C) Selective precipitation of very high molecular weight (vHMW) DNA fibers by condensation into a single "cocoon" near the interface between 75% ethanol and CsCl solution. The 75% ethanol was layered onto pooled CsCl gradient DNA bands (see Methods). The hazy cloud extending up from the interface and partially obscuring the forming cocoon is a precipitate of salts, low-molecular-weight DNA fragments, RNA and other contaminants that will be decanted upon completion of vHMW DNA precipitation. Any remainder will be removed by a 70% EtOH wash. (D) Examples of fully condensed cocoons in 70% ethanol. [file 1756-0500-4-499-S3.PDF]

# Stationary phase culture

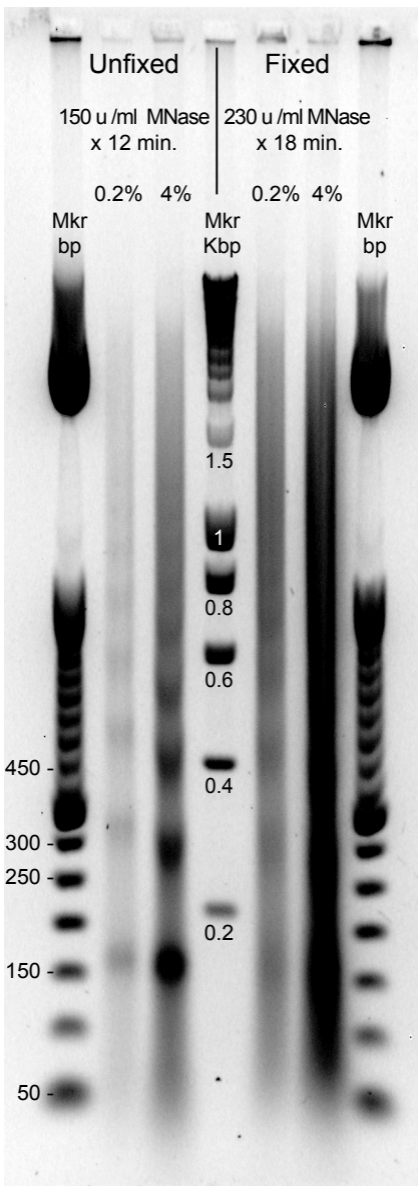

Supplement: Additional file 4 — Gel-electrophoretic analysis of DNA from MNase-digested nuclei of unfixed and fixed stationary-phase fission yeast cells. Half of the cells in a stationary-phase culture were fixed with formaldehyde, then both portions of the culture were cryo-fixed and ground to liberate nuclei. Washed nuclei were incubated in parallel at 25°C for the indicated times in INCA buffer, at the indicated MNase concentrations. The percentage labels denote the portion of the respective digest sample loaded into a given lane. "Mkr bp": 50-bp DNA-size-marker ladder. "Mkr Kbp": 0.2- to 10-kbp DNA-size-marker ladder. [file 1756-0500-4-499-S4.PDF]
